# Optimization of Textural and Structural Properties of Carbon Materials for Sodium Dual-Ion Battery Electrodes

**DOI:** 10.3390/molecules30112439

**Published:** 2025-06-02

**Authors:** Ignacio Cameán, Belén Lobato, Rachelle Omnée, Encarnación Raymundo-Piñero, Ana B. García

**Affiliations:** 1 Instituto de Ciencia y Tecnología del Carbono, INCAR-CSIC, Francisco Pintado Fe 26, 33011 Oviedo, Spain; belen@incar.csic.es; 2 CNRS, CEMHTI UPR 3079, University Orléans, 45071 Orléans, France; rachelle.omnee@cnrs-orleans.fr (R.O.); encarnacion.raymundo@cnrs-orleans.fr (E.R.-P.)

**Keywords:** carbon materials, anode, cathode, sodium dual-ion batteries

## Abstract

Sodium dual-ion batteries combine economic and environmental benefits by using carbon materials in both electrodes and sodium compounds in the electrolyte. Among other factors, their successful implementation for energy storage relies on optimization of the properties of the carbon electrode materials. To this end, carbon materials with a wide range of textural and structural properties were prepared by simply heat treating a single porous carbon in the absence or presence of a low-cost highly effective iron-based catalyst. These materials were investigated as anode or cathode in the sodium dual-ion batteries by prolonged galvanostatic cycling. The optimal textural and structural properties for carbon materials to achieve the best performance as electrodes in sodium dual-ion batteries were identified as having a high degree of graphitic structural order combined with minimal microporosity in the cathode and a non-graphitic structure with a layer spacing of around 0.37 nm and moderate microporosity in the anode.

## 1. Introduction

The concept of dual-ion batteries (DIBs) was first described in a patent by McCullough et al. in 1989 [1]. DIB functioning is based on the storage by different mechanisms of both cations and anions from the electrolyte salt in the anode and the cathode, respectively, during the charge; while the reverse process occurs in the discharge. Because of the crucial role played by the electrolyte, a wide variety of lithium or sodium salts dissolved in mixtures of organic carbonates has been studied for these batteries [2,3,4,5], hence they are named lithium dual-ion batteries (Li-DIBs) or sodium dual-ion batteries (Na-DIBs). DIBs are a promising alternative to lithium-ion batteries (LIBs) because of their expected lower energy cost, given that the lithium compounds of the cathode, responsible for the highest portion of LIBs’ energy cost [6], can be replaced here by carbon materials, which are mass-produced from a wide variety of precursors and are characterized by low cost, availability and a unique combination of properties that can be easily tailored to specific applications [7].

Among DIBs, Na-DIBs combine the benefits of using carbon materials in the electrodes and electrolytes based on sodium compounds, which are of greater abundance, more equitably distributed in the earth, of lower cost and of a more environmentally-friendly nature than the lithium compounds. Because DIB energy density is determined by the scope of the anion intercalation in the cathode at the highest working potential possible, the performance of different anions, such as BF_4_^−^, ClO_4_^−^ and PF_6_^−^, which clearly overcome the others, has been studied using different concentrations of sodium salts dissolved in mixtures of organic carbonates as electrolytes [2,3,4,5]. In these studies, many different carbon materials, including oil-derived synthetic graphite, expanded graphite, amorphous ordered mesoporous carbons, graphitic biogas-derived nanofibers and graphitized carbon xerogels, have been used as cathode materials for Na-DIBs [8,9,10,11,12,13,14]. Given specific electrolyte composition and working potential, it was found that anions intercalate/de-intercalate more effectively in graphitic materials with a higher degree of crystallinity because of more accessible pathways and fast diffusivity [15], resulting in larger capacities, improved cycling stability and good rate performance [3,13,14]. However, graphite cathode capacity is usually limited to approximately 100 mAh g^−1^ because of the loss of stability of the electrolytes beyond 5 V [3,8]; therefore, a significant number of the electrochemically active centres in the graphite lattice could remain unapproachable.

Taking advantage of the large number of studies devoted to sodium-ion batteries (SIBs), research on materials for Na-DIB anodes has primarily focused on hard carbons (no structurally ordered carbon materials containing small graphitic domains), which have been identified as the most promising materials for the storage of Na^+^ ions due to, among other reasons, the low sodium storage potential, capacity and variety of precursors (fossil fuels, biomass, polymeric carbons) and synthetic routes that result in materials with different and tailorable properties [16,17,18,19]. In this context, polymer-based, walnut shell-derived, fuel-derived and doped hard carbons among many others, have been investigated as anodes for Na-DIBs [3,7,19,20,21].

Nevertheless, despite the fact that research on Na-DIBs has increased exponentially in this decade, the optimum properties of carbon materials for high performance of both anodes and cathodes in preventing limited capacity and large irreversible capacity losses during the first cycles that negatively affect the performance and cycling stability of the battery have not yet been established. In addition, reproducibility of the carbon material properties is challenging because of the variable composition of their usual precursors, such as biomass and fossil fuel wastes.

Based on these considerations, carbon materials with varying surface areas, microporosity and degrees of structural order were prepared from the same precursor at a moderate temperature by using a low-cost iron-based catalyst that assures materials reproducibility. The carbons were investigated as anode and cathode materials for Na-DIBs through prolonged galvanostatic cycling at various electric current densities. This work is focused on determining the optimal textural and structural properties of the carbon materials for this specific application, considering their influence on electrode reversible capacity, irreversible capacity in the first cycles, capacity retention along cycling and coulombic efficiency. The mechanisms of ion storage in the carbon materials are examined.

## 2. Results and Discussion

### 2.1. Structural, Morphological and Textural Properties of the Carbon Materials

**Figure 1:**
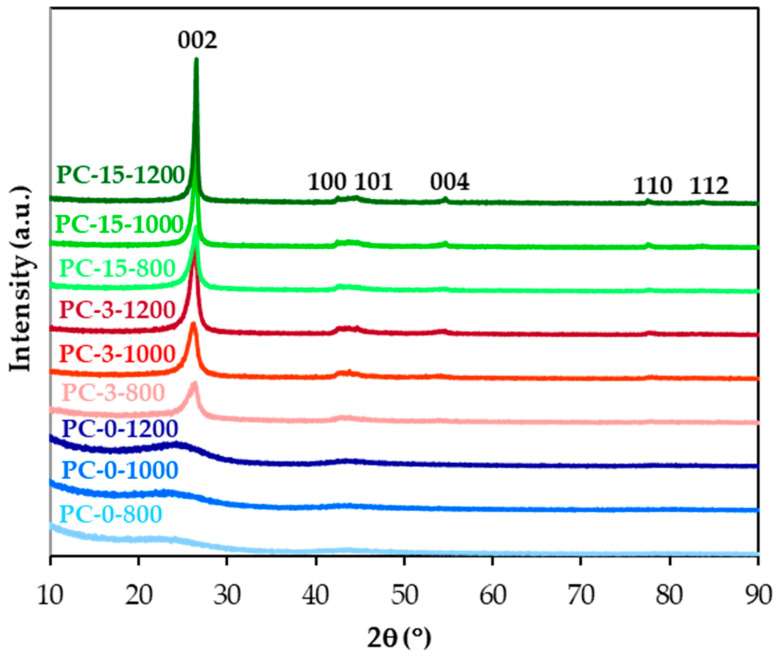
XRD diffractograms of the carbon materials.

Figure 1 shows the X-ray diffractograms of the carbon materials that were prepared and named as described in Section 3.1. As can be seen, increasing the carbonization temperature and, particularly, impregnation with a solution of an iron compound leads to sharper and more defined peaks. Moreover, the (002) peak at ~27° slightly shifts to the higher 2θ, and the broad peak around 43–44°, attributed to (100) and (101) reflexions, as well as the (004) at ~54°, the (110) at ~77–78°, and the (112) at 83–84° peaks become clearly observable. These effects are due to the increase of the graphite-type structural order of the materials, as shown by the gradual decrease in the interlayer distance, d_002_ and the increase in the main crystalline size along the c-axis, L_c_ (Table 1), that were calculated by applying the Bragg’s equation and the Scherrer formula, respectively (Section 3.2). Therefore, the PC-15-1200 material that was produced at 1200 °C in the presence of the largest amount of iron, which is known to be an excellent graphitization catalyst of carbon materials [22] in general and coke in particular [23], shows the most ordered carbon structure, with a d_002_ of 0.3366 nm and a L_c_ of 24.3 nm. For comparison, the average number of parallel stacked graphene layers (N), a numerical estimation of the graphite-type degree of structural order, was calculated using the equation N = L_c_/d_002_, and the data are presented in Table 1. This degree follows the order PC-15-1200 > PC-15-1000 >> PC-15-800 > PC-3-1200 >> PC-3-1000 > PC-3-800 >> PC-0-1200~PC-0-1000~PC-0-800. That is, at a given temperature, it increases significantly with the amount of iron, clearly highlighting the primary role of this element. For example, the N value of PC-15-1200 is 2.7 and up to 17.6 times higher than those values calculated for PC-3-1200 and PC-0-1200, respectively. According to the classification of Rosalind Franklin [24], only the iron-doped based materials can be considered graphitic, i.e., those with d_002_ values in the range of 0.3354–0.3400 nm, although PC-3-1000 and PC-3-800 fall slightly outside of this range.

**Figure 2:**
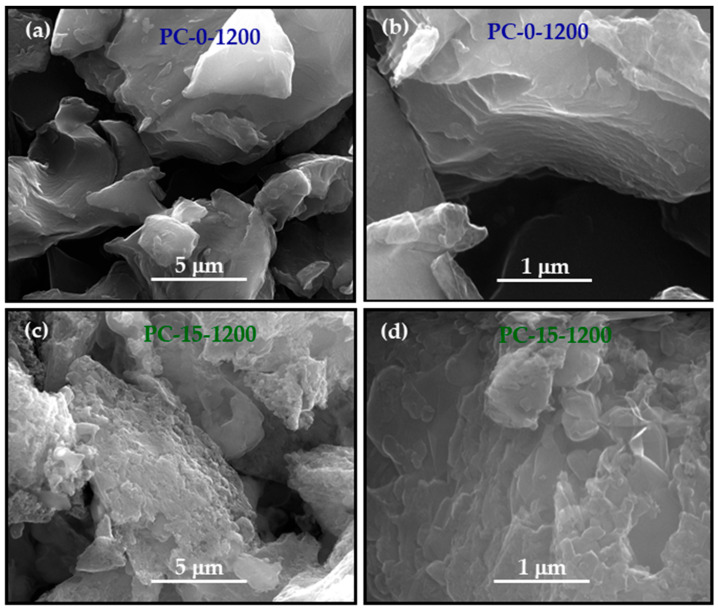
SEM images of (**a**,**b**) PC-0-1200 and (**c**,**d**) PC-15-1200 materials.

The increase in the structural ordering of the materials induced by iron becomes graphically evident when comparing the SEM micrographs of PC-0-1200 (non-graphitic) and PC-15-1200 (graphitic) in Figure 2, in which the development of quasi-parallel stacks of graphene layer in the latter is observed. Additionally, this element causes changes in the surface morphology of the materials, resulting in a rougher surface.

The textural parameters of the PC precursor and the carbon materials prepared, calculated from the corresponding N_2_ adsorption-desorption isotherms (see Figure S1), are reported in Table 2. The materials exhibit a type IV isotherm, based on the IUPAC classification [25], with a hysteresis loop due to the presence of mesopores. As expected, both the specific surface area and the pore volume tend to decrease with the increase in the degree of graphite-type structural order, i.e., with increasing treatment temperature/amount of iron. However the reduction in the values of these textural parameters, which can be mainly ascribed to the loss of microporosity given that mesoporosity does not follow the same trend, reaches a lower limit regardless of any further increase in the material structural order. Thus, PC-15-1000 and PC-15-1200 with N values of ~54 and 72, respectively (Table 1), display essentially the same S_BET_ (94 m^2^ g^−1^) and V_p_ (0.27–0.30 m^3^ g^−1^) values (Table 2). The materials prepared in the presence of iron show a significant development of mesoporosity with maxima values in the range of 0.44–0.55 m^3^ g^−1^.

### 2.2. Carbon Materials: Performance as Cathodes

**Figure 3:**
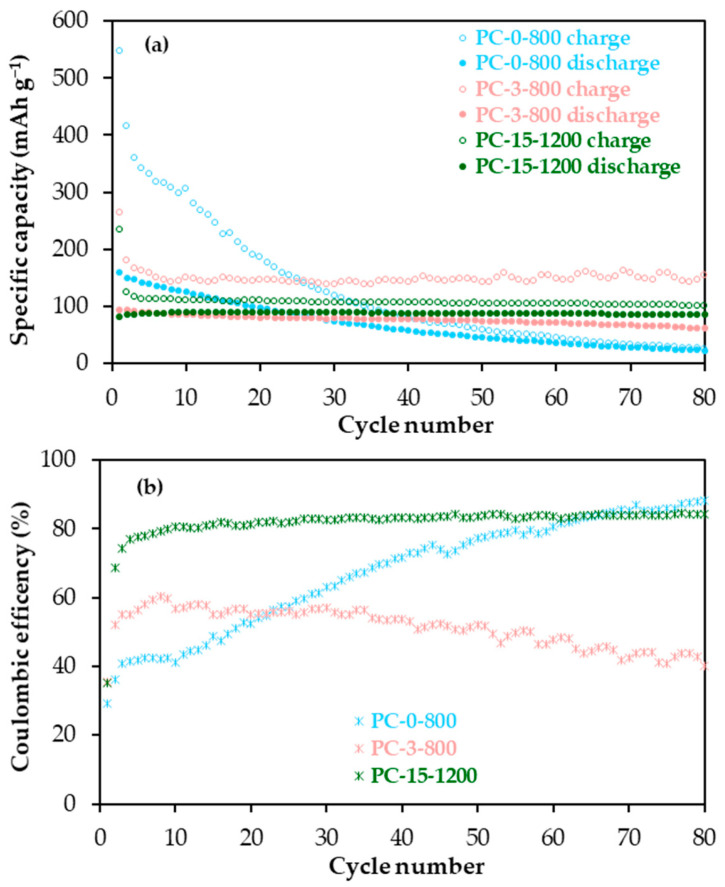
(**a**) Specific capacity and (**b**) coulombic efficiency versus cycle number plots of the PC-0-800, PC-3-800 and PC-15-1200 cathodes at 50 mA g^−1^, in the 2.90–5.00 V vs. Na/Na^+^ potential range.

The PC-0-800 (non-graphitic, high porosity), PC-3-800 (low graphitic, medium porosity) and PC-15-1200 (graphitic, low porosity) materials with very different structural and textural properties were selected to study the influence of these properties on their performance as cathodes for dual-ion batteries. Figure 3 shows the specific capacity and the coulombic efficiency (discharge/charge capacity) against the cycle number plots from the galvanostatic cycling of these materials, in the 2.90–5.00 V vs. Na/Na^+^ range, at a current density of 50 mA g^−1^ (the electrochemical parameters are compiled in Table S1).

Among the materials tested, PC-15-1200 shows the best electrochemical performance as a cathode (Figure 3). Specifically, this material provides a charge capacity (PF_6_^−^ anion storage) of ~100 mAh g^−1^ after 80 charge-discharge cycles, with remarkable cycling stability (charge-discharge capacity retentions of 82–100% in the 2–80 cycle range). For comparison, a slightly higher charge capacity was determined for PC-3-800 throughout cycling. However, this material exhibits much lower coulombic efficiency and a slow but continuous capacity decline, mainly in terms of discharge. As for PC-0-800, although it initially shows greater capacity than both PC-3-800 and PC-15-1200, this cathode experiences a rapid and abrupt drop in capacity already from the initial cycles, so that the value at the end of cycling is as low as 25 mAh g^−1^ (Figure 3a). Moreover, this material displays the lowest coulombic efficiency during the first 20 cycles (Figure 3b); in this cycling period, charge and discharge capacities in the 548–186 mAh g^−1^ and 160–97 mAh g^−1^ ranges, respectively, were determined. Coulombic efficiency values below 100% are primarily ascribed to the continuous decomposition of the electrolyte on the surface of carbon-based cathodes at high potentials [26,27,28]. This effect, which may cause electrode cycling destabilization and consequently capacity fading, tends to increase with the surface area of the electrode active material (Table 2) as can be seen by comparing the differential capacity versus potential plots for cycle 12 (just before the dramatic capacity fading in PC-0-800) of the materials under discussion shown in Figure S2, specifically regarding the peak (charge capacity) around 5 V vs. Na/Na^+^, whose intensity follows the sequence PC-0-800 (1591 m^2^ g^−1^) >> PC-3-800 (771 m^2^ g^−1^) >> PC-15-1200 (94 m^2^ g^−1^). Additionally, based on previous studies [29], the trapping of PF_6_^−^ anions in materials such as PC-0-800 with a significant volume of micropores (Table 2) cannot be ruled out, thus helping to explain the drastic drop in discharge capacity during cycling (Figure 3a), and therefore contributing to lower coulombic efficiency.

**Figure 4:**
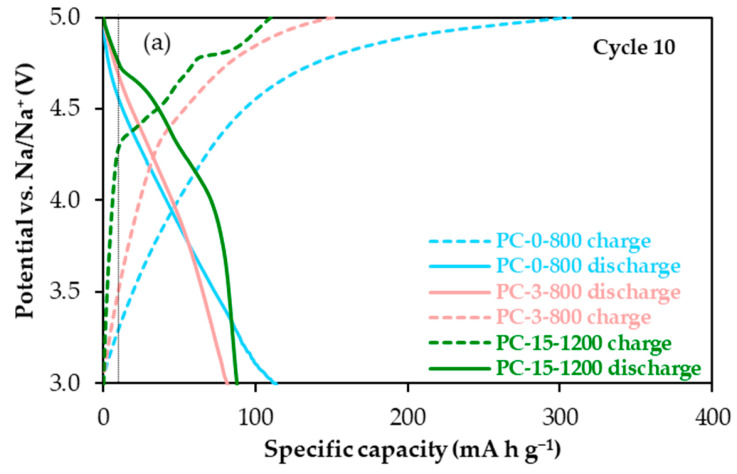

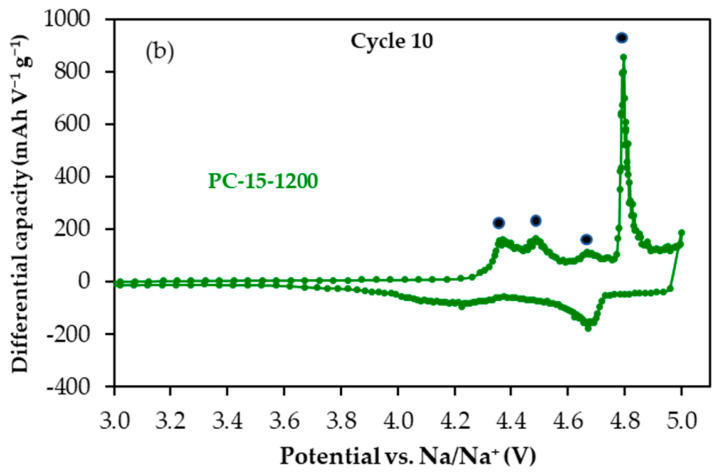
(**a**) Potential versus specific capacity plots of PC-0-800, PC-3-800 and PC-15-1200 cathodes and (**b**) differential capacity versus potential plot of PC-15-1200 cathode for cycle 10 at 50 mA g^−1^ in the 2.90–5.00 V vs. Na/Na^+^ potential range.

It becomes evident from these results that the textural properties of the materials, particularly the microporosity, will determine the cathode coulombic efficiency and therefore, its cycling stability, i.e., capacity recovery. Nevertheless, these parameters do not fully explain the differences in material capacity, specifically during the first cycles and in terms of discharge capacity, which is not directly affected by electrolyte decomposition and, at a first glance, appears to be favoured by material porosity. For example, in cycle 10, PC-0-800 provided 127 mAh g^−1^, while values in the 86–89 mAh g^−1^ range were obtained for PC-3-800 and PC-15-1200 (Figure 3a). To delve deeper into this issue, the potential profiles (potential vs. Na/Na^+^) from the cycling of these materials were studied in detail. As an example, those for cycle 10, together with the differential capacity plot corresponding to the PC-15-1200 material, are shown in Figure 4.

The potential profile of PC-15-1200 shows four and two plateaux in the charge (PF_6_^−^ intercalation) and discharge (PF_6_^−^ de-intercalation), respectively (Figure 4a). Furthermore, as seen in the corresponding differential capacity versus potential plot in Figure 4b, the intercalation peaks are sharper and more defined, indicating that this process is kinetically more favourable than de-intercalation [27]. Anion intercalation in this material occurs at approximately 4.37, 4.50, 4.68 and 4.80 V vs. Na/Na^+^; while the de-intercalation peaks appear at 4.68 and 4.22 V vs. Na/Na^+^. In contrast to PC-15-1200, no plateau regions, i.e., no staged mechanisms, are observed in the potential profiles of PC-0-800 and PC-3-800. These profiles only exhibit sloping regions in both the charge and discharge processes, suggesting that, provided with the above-mentioned decomposition at the highest potential, the PF_6_^−^ anion storage in these materials goes through a similar single mechanism based on adsorption on the surface (defects and pores) similar to that proposed for the storage of Na^+^ ions in non-graphitic carbons (hard carbons) [30,31]. Consequently, the cathode capacity initially tends to increase with the active material microporosity, i.e., surface defects, as discussed above. Moreover, the onset potential for the PF_6_^−^ anion de-storage (discharge) follows the sequence PC-0-800 < PC-3-800 < PC-15-1200, with values of 4.55, 4.66 and 4.75 V vs. Na/Na^+^, respectively (in Figure 4a, a discharge capacity of 10 mAh g^−1^ was assumed for the determination of the onset potential for anion de-storage). These findings imply that less energy is initially required for anion de-storage in the PC-0-800 cathode than in the other two, which may somehow account for its higher discharge capacity during the first cycles (Figure 3a).

**Figure 5:**
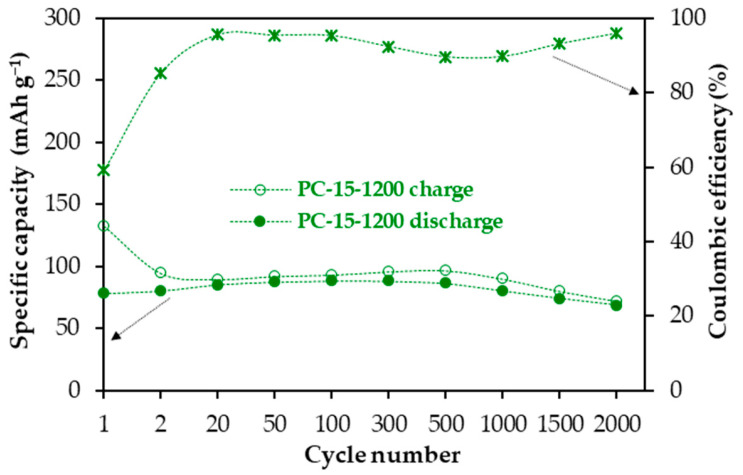
Specific capacity and coulombic efficiency versus cycle number plot of PC-15-1200 cathode at 500 mA g^−1^ in the 2.90–5.00 V vs. Na/Na^+^ potential range.

Based on the above results, the increase in the graphitic degree of structural order of the cathode material, which in turn leads to a decrease in microporosity, improves its performance in terms of capacity, stability and coulombic efficiency. Even so, PC-15-1200 material, despite having the best overall features, still shows a relatively low coulombic efficiency at a current density of 50 mA g^−1^ (approximately 81% after stabilization; Figure 3a). PC-15-1200 was tested as a cathode under prolonged cycling at 500 mA g^−1^ in the 2.90–5.00 V vs. Na/Na^+^ range for 2000 cycles, and the plot of specific capacity and coulombic efficiency versus cycle number is shown in Figure 5. As observed, higher current densities, i.e., higher cycling rates, which imply shorter electrode/electrolyte contact times at high potentials and, therefore, decrease the scope of electrolyte decomposition on the electrode surface, lead to a significant increase in coulombic efficiency. For comparison, a coulombic efficiency of ~96% was measured after electrode stabilization (cycle 20, Figure 5) versus a value of ~81% at the lower current density of 50 mA g^−1^ (Figure 3b). Moreover, increasing the applied current density tenfold does not significantly compromise the capacity delivered by the PC-15-1200 cathode, which also shows remarkable capacity retention over prolonged cycling with values around 80% in the 20–2000th cycle range.

In summary, a high degree of structural order combined with minimal microporosity are the optimal characteristics for a carbon material to achieve the best performance as a cathode in a sodium dual-ion battery.

### 2.3. Carbon Materials: Performance as Anodes

**Figure 6:**
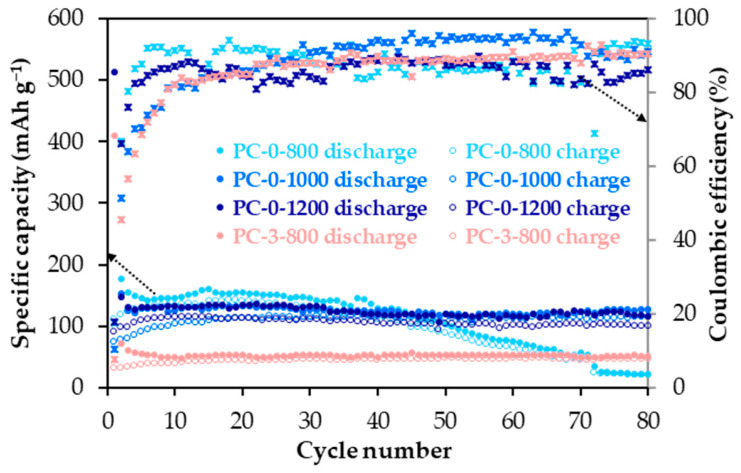
Specific capacity and coulombic efficiency versus cycle number plots of PC-0-800, PC-0-1000, PC-0-1200 and PC-3-800 anodes at 50 mA g^−1^ in the 2.10–0.01 V vs. Na/Na^+^ potential range.

The following materials with different structural and textural characteristics, namely PC-0-800 (non-graphitic, high porosity), PC-0-1000 (non-graphitic, high porosity), PC-0-1200 (non-graphitic, medium porosity) and PC-3-800 (low graphitic, medium porosity) materials were chosen to study the influence of these properties on their performance as anodes. Plots of specific capacity and coulombic efficiency (charge/discharge capacity) versus cycle number for these materials in the 2.10–0.01 V vs. Na/Na^+^ potential range at a current density of 50 mA g^−1^ are shown in Figure 6 (the electrochemical parameters are compiled in Table S2).

Among the materials tested, those that are non-graphitic (d_002_ > 0.3400 nm) and possess a larger volume of micropore volume provide significantly higher capacity (Table 1 and Table 2, Figure 6). In fact, there is a clear trend of increasing capacity with both the interlayer spacing and microporosity. For comparison, the discharge capacity (Na^+^ ions storage) values of approximately 154, 133, 134 and 53 mAh g^−1^ are displayed by PC-0-800, PC-0-1000, PC-0-1200 and PC-3-800, respectively, at cycle 20. However, although the material microporosity seems to positively influence their anodic capacity, the associated large surface area negatively impacts coulombic efficiency, with values below 100%, suggesting electrolyte decomposition at the anode material surface that eventually, can lead to capacity fading, as in the case of PC-0-800 with the highest surface area (Table 2). Therefore, an adequate balance between material structural order and microporosity must be reached to achieve the best possible electrochemical performance as an anode. To gain further insight into this issue, the potential profiles (potential vs. Na/Na^+^ against capacity, see Figure S3) from galvanostatic cycling at a current density of 50 mA g^−1^,were analysed in detail for all selected anode materials. At a first glance, no remarkable differences are observed in the potential profile shapes of these materials, particularly among the non-graphitic ones, i.e., the same Na^+^ ions’ storage mechanism. Upon descending potential, two distinct zones appear, a sharply sloping curve followed by a more or less sloping plateau (Figure S3). According to the adsorption-intercalation model, the electrochemical storage of the Na^+^ ions in disordered carbon materials occurs through two processes, namely, (i) adsorption on the surface (defects such as micropores and/or pores) and (ii) insertion between the aromatic carbon layers of the small graphitic domains [30,31]. The sloping zone of the potential profile above 0.1 V is related to the adsorption, while the plateau below 0.1 V is attributed to insertion. To analyse the differences among materials, the proportional distribution of the discharge capacity (Na^+^ storage) between these two zones were calculated for cycles 10, 25 and 50 based on the potential profiles in Figure S3. The resulting data are given in Table 3.

As seen in Table 3, the Na^+^ ion storage capacity coming from adsorption on the surface is highly predominant, and it tends to increase with material microporosity, i.e., the amount of surface defects (Table 2). Accordingly, PC-0-800 with the largest micropore volume (0.65 cm^3^ g^−1^) initially shows the highest discharge capacity contribution above 0.1 V. However, due to its correspondingly high surface area, which, as mentioned above, can promote electrolyte decomposition, this capacity contribution significantly declines with cycling. On the contrary, this contribution remains basically constant for PC-0-1200 and PC-3-800 with much lower microporosity and, therefore, surface area. The discharge capacity contribution at below 0.1 V follows the sequence PC-3-800 < PC-0-800 ≈ PC-0-1000 < PC-0-1200. When comparing this trend and the material XRD parameters listed in Table 1, it becomes evident that there is an optimal carbon layer spacing value of around 0.37 nm (as observed for PC-0-1200) for efficient insertion of the Na^+^ ions in these materials. This result agrees with that predicted for carbon materials by theoretical calculations [32,33].

Therefore, a non-graphitic structure and moderate microporosity are the optimal characteristics for a carbon material to achieve the best performance as an anode in a sodium dual-ion battery.

## 3. Materials and Methods

### 3.1. Carbon Materials

A commercial porous carbon (PC) obtained from a carbon coke by activation with KOH (Sigma-Aldrich, Darmstadt, Germany), was heat treated between 800 and 1200 °C in the presence and absence of iron. The raw carbon (PC) was firstly impregnated with a dissolution of iron nitrate Fe(NO_3_)·9H_2_O (Sigma-Aldrich, Darmstadt, Germany) in ethanol followed by (i) heat treatment in a tubular furnace under 150 mL min^−1^ N_2_ flow at 800, 1000 and 1200 °C for 1 h at a heating rate of 10 °C min^−1^, (ii) washing with HCl (10%) and H_2_O to remove metal particles and finally (iii) dried at 80 °C overnight. Iron concentrations of 3 and 15 mmol per gram of PC were used [34]. Materials in the absence of Fe were also prepared from PC by the same procedure. The resulting carbon materials were designated based on the iron added and the treatment temperature, such as for example PC-0-800 (no iron added, 800 °C) or PC-3-1000 (3 mmol Fe/g PC, 1000 °C).

### 3.2. Characterization Techniques

For the study of the porous texture of the materials, N_2_ adsorption-desorption isotherms were carried out at at −196 °C in ASAP 2020 (Micromeritics, Norcross, GA, USA). Prior to these measurements, the materials were outgassed under vacuum overnight at 120 °C. The specific surface area (S_BET_) and the micro-(V_micro_) and mesopore (V_meso_) volumes were calculated from the isotherms by applying the Brunauer–Emmett–Teller (BET) [35] and the Dubinin–Raduskevich equations [36], respectively. The total pore volume (V_p_) was determined from the amount of nitrogen adsorbed at the saturation point (p/p_o_ = 0.99).

X-ray diffraction (XRD) patterns of the materials were recorded using a Bruker D8 Advance diffractometer (Billerica, MA, USA), operating at 40 kV and 40 mA with a Cu-Kα radiation (λ = 0.15406 nm) as the source. Data were collected from 10 to 90° over a 2θ range with a step size of 0.02° and an interval of 3 s per step, as described elsewhere [37]. A silicon standard was used to correct the broadening of the diffraction peaks caused by instrumental factors. The interlayer spacing (d_002_) and the mean crystallite size along the perpendicular c-axis (L_c_) were calculated from the (002) diffraction peak in the corresponding XRD pattern by applying Bragg’s equation and the Scherrer formula with a K value of 0.9, respectively [38]. These parameters were used to evaluate the degree of graphitic structural order (degree of graphitization) of the materials.

The morphology of the materials was examined by scanning electron microscopy (SEM). The images were acquired in a Quanta FEG 650 (FEI Company, Hillsboro, OR, USA) instrument using an acceler-ating voltage of 20 kV and an Everhart-Thornley secondary electron detector (ETD) equipped with energy-dispersive X-ray spectroscopy (EDX). EDX analysis was used to confirm the removal of the residual metal particles from the iron-doped materials (see Figure S4). Samples were attached to an aluminium tap using conductive double-sided adhesive carbon tape. No further coating was used.

### 3.3. Electrode Preparation, Cells Assembly and Electrochemical Measurements

Two-electrode (working, WE, and counter, CE, electrodes) Swagelok-type cells were used for the electrochemical measurements. WEs containing 70 wt.% of the corresponding carbon material (active material), 20 wt.% of sodium carboxymethylcellulose (NaCMC, Sigma-Aldrich, Darmstadt, Germany, Mw ~700,000) binder and 10 wt.% of carbon black (Super C65, Imerys Graphite & Carbon, Paris, France) conductive additive were prepared as follows: (i) the NaCMC was dissolved in 20 mL of de-ionized water by mechanical stirring (IKA Overhead Stirrer Eurostar20, Barcelona, Spain) for 1 h at 2000 rpm to obtain a 1.0 wt.% solution; (ii) the C65 was mixed into the NaCMC solution while keeping stirring (15 min, 1000 rpm), to attain a homogeneous dispersion; (iii) the active material was then added gently to form a slurry, which was stirred (1 h, 4000 rpm) to ensure good homogenization as well as the absence of agglomerates; (iv) the slurry was tape-casted on aluminium foil (99.3 wt.% purity, 20 µm thickness, supplied by Hohsen Corporation, Osaka, Japan) at 50 °C, using a standard doctor blade with a gap of 350 µm and a motorized/automatic film applicator Elcometer 4340, provided with a perforated heated vacuum table; (v) the electrode tape was maintained over the applicator table under vacuum at 80 °C for 1 h; (vi) electrodes of 12 mm diameter were cut from the tape using a manual punching machine; and (vii) these electrodes were subjected to an additional drying step at 120 °C for 2 h under vacuum in a glass oven (B-585, Büchi) and stored in a glove box (MBraun) under an Ar atmosphere, with oxygen and water contents below 0.1 ppm. The average active mass load of the electrodes was in the range of 1.5 mg cm^−2^.

Metallic sodium (≥99.9 wt.% purity from Merck/Sigma-Aldrich, Darmstadt, Germany) discs of 12 mm di-ameter were employed as CE. Two micro-fibre glass discs WHATMAN (Buckinghamshire, UK) GF/A of 12 mm diameter, soaked with 250 µL of electrolyte, were placed between WE and CE. Based on previous works [8,11,13,14,18,19,39], an electrolyte consisting of 2.0 M solution of sodium hexafluorophosphate (NaPF_6_), salt (>99% purity, Sigma-Aldrich, Darmstadt, Germany) in ethylene carbonate (EC, 99.0% purity, Merck, Darmstadt, Germany) and ethyl methyl carbonate (EMC, ≥99.9% purity, Sigma-Aldrich, Darmstadt, Germany), 1:1 (*w*:*w*) was used. The assembly of the cells was conducted in the glove box, and their initial potential was in the 2.8–3.0 V vs. Na/Na^+^ range.

The electrochemical tests were carried out in a Biologic Battery Cycler BCS810 (Biologic Science Instruments, Seyssinet-Pariset, France). The two-electrode cells were subjected to galvanostatic cycling (discharge/charge cycles), in the potential ranges of 2.10–0.01 V vs. Na/Na^+^ and 2.90–5.00 V vs. Na/Na^+^ for the anode and the cathode, respectively, at current densities of 50 and 500 mA g^−1^.

## 4. Conclusions

Carbon materials with a wide range of textural and structural properties were obtained by simply heat treating a porous carbon in the absence or in the presence of a low-cost highly effective iron-based catalyst. The diversity of surface area, microporosity and degree of structural order characteristics achievable from a single precursor enabled us to identify the properties of carbon materials that optimize their performance as anodes or cathodes in sodium dual-ion batteries. Among the carbon material properties, a high degree of graphite-like structural order and low microporosity were identified as key factors that contribute to optimal cathodic performance. Although an increase in material microporosity may initially improve the extent of anion storage through an adsorption process, the associated increase in surface area gives rise to greater electrolyte decomposition, which ultimately causes electrode destabilization and a continuous loss of capacity during cycling. For the anode, a non-graphitic structure and a discrete microporosity have been demonstrated to be the optimal properties to achieve the best material performance. Since the Na^+^ ion storage mechanism in these materials is a combination of insertion between the graphene layers and adsorption on the surface, an adequate balance between material structure and microporosity with an optimal carbon layer spacing of around 0.37 nm was determined.

From the conclusions of this study, future works will be focused on the design and production of full sodium dual-ion batteries with electrodes based on carbon materials with optimized textural and structural properties.

## Figures and Tables

**Table 1:** Interlayer spacing, d_002_, mean crystallite size along the c-axis, L_c_, average number of parallel stacked graphene layers, N (L_c_/d_002_) and Franklin’s classification of the carbon materials.

Material	d_002_ (nm)	L_c_ (nm)	N	Classification
PC-0-800	0.3777	2.1	5.6	Non-graphitic
PC-0-1000	0.3737	1.5	4.0	Non-graphitic
PC-0-1200	0.3663	1.5	4.1	Non-graphitic
PC-3-800	0.3417	6.0	17.6	Low graphitic
PC-3-1000	0.3417	6.7	19.6	Low graphitic
PC-3-1200	0.3397	9.0	26.5	Graphitic
PC-15-800	0.3388	9.9	29.2	Graphitic
PC-15-1000	0.3371	18.3	54.3	Graphitic
PC-15-1200	0.3366	24.3	72.2	Graphitic

**Table 2:** Textural parameters of the PC precursor and the carbon materials prepared: BET surface area (S_BET_), mesopore volume (V_meso_), micropore volume (V_micro_) and total pore volume (V_p_).

Material	S_BET_ (m^2^ g^−1^)	V_meso_ (cm^3^ g^−1^)	V_micro_ (cm^3^ g^−1^)	V_p_ (cm^3^ g^−1^)
PC	1560	0.21	0.62	0.82
PC-0-800	1591	0.16	0.65	0.81
PC-0-1000	1332	0.21	0.48	0.69
PC-0-1200	991	0.15	0.36	0.51
PC-3-800	771	0.36	0.28	0.64
PC-3-1000	336	0.44	0.13	0.57
PC-3-1200	213	0.55	0.08	0.47
PC-15-800	216	0.39	0.10	0.54
PC-15-1000	94	0.24	0.03	0.27
PC-15-1200	94	0.26	0.04	0.30

**Table 3:** Discharge capacity distribution above and below 0.1 V from the cycling of PC-0-800, PC-0-1000, PC-0-1200 and PC-3-800 materials at 50 mA g^−1^.

Cycle	PC-0-800 (mAh g^−1^)		PC-0-1000 (mAh g^−1^)		PC-0-1200 (mAh g^−1^)		PC-3-800 (mAh g^−1^)	
	>0.1 V	<0.1 V	>0.1 V	<0.1 V	>0.1 V	<0.1 V	>0.1 V	<0.1 V
10	114	28	97	29	89	43	35	14
25	126	27	106	26	89	44	37	13
50	92	19	97	23	81	38	40	14

## Data Availability

The original contributions of the study are included in the article, further inquiries can be directed to the corresponding authors.

## Supplementary Materials

The following supporting information can be downloaded at: https://www.mdpi.com/article/10.3390/molecules30112439/s1; Figure S1: Nitrogen adsorption-desorption isotherms: (a) PC, PC-0-800, PC-0-1000, PC-0-1200, (b) PC-3-800, PC-3-1000, PC-3-1200 and (c) PC-15-800, PC-15-1000, PC-25-1200; Figure S2: Differential capacity versus voltage plot for 12th cycle of PC-0-800, PC-3-800 and PC-15-1200 cathodes at 50 mA g^−1^ in the 2.9-5.0 V range; Figure S3: Potential versus discharge capacity plots of PC-0-800, PC-0-1000, PC-0-1200 and PC-3-800 anodes for cycles 10, 25 and 50, in the 2.10–0.01 V vs. Na/Na+ potential range; Figure S4: EDX analysis of PC-3-800 and PC-15-1200 materials. Table S1: Specific charge capacity (Ccharge) in the 1st, 2nd, 10th, 20th, 50th and 80th cycles and corresponding coulombic efficiency (CE), and irreversible capacity in the 1st cycle (Cirr) from the galvanostatic cycling in the 2.90–5.00 V vs. Na/Na+ potential range at 50 mA g^−1^ for PC-0-800, PC-3-800 and PC-15-1200 material-based cathodes; Table S2: Specific discharge capacity (Cdis) in the 1st, 2nd, 10th, 20th, 50th and 80th cycles and corresponding coulombic efficiency (CE), and irreversible capacity in the 1st cycle (Cirr) from the galvanostatic cycling in the 2.10–0.01 V vs. Na/Na+ potential range at 50 mA g^−1^ for PC-0-800, PC-0-1000, PC-0-1200 and PC-3-800 material-based anodes.
