# Supplementary material for: Optimization of Textural and Structural Properties of Carbon Materials for Sodium Dual-Ion Battery Electrodes
Source: Molecules. 2025 Jun 2;30(11):2439. doi: 10.3390/molecules30112439 (PMC12156141; doi:10.3390/molecules30112439)
Supplement: Supplementary file 1 [file molecules-30-02439-s001.zip › molecules-3641549-supplementary.pdf]

Article

Optimization of Textural and Structural Properties of Carbon Materials for Sodium Dual-Ion Battery Electrodes

Ignacio Cameán [1,*], Belén Lobato [1], Rachelle Omnée [2], Encarnación Raymundo-Piñero [2] and Ana B. García [1,*]

1 Instituto de Ciencia y Tecnología del Carbono, INCAR-CSIC, Francisco Pintado Fe 26, 33011 Oviedo, Spain; belen@incar.csic.es

2 CNRS, CEMHTI UPR 3079, University Orléans, 45071 Orléans, France; rachelle.omnee@cnrs-orleans.fr (R.O.); encarnacion.raymundo@cnrs-orleans.fr (E.R.-P.)

* Correspondence: icamean@incar.csic.es (I.C.); anabgs@incar.csic.es (A.B.G.)

SUPPLEMENTARY MATERIAL

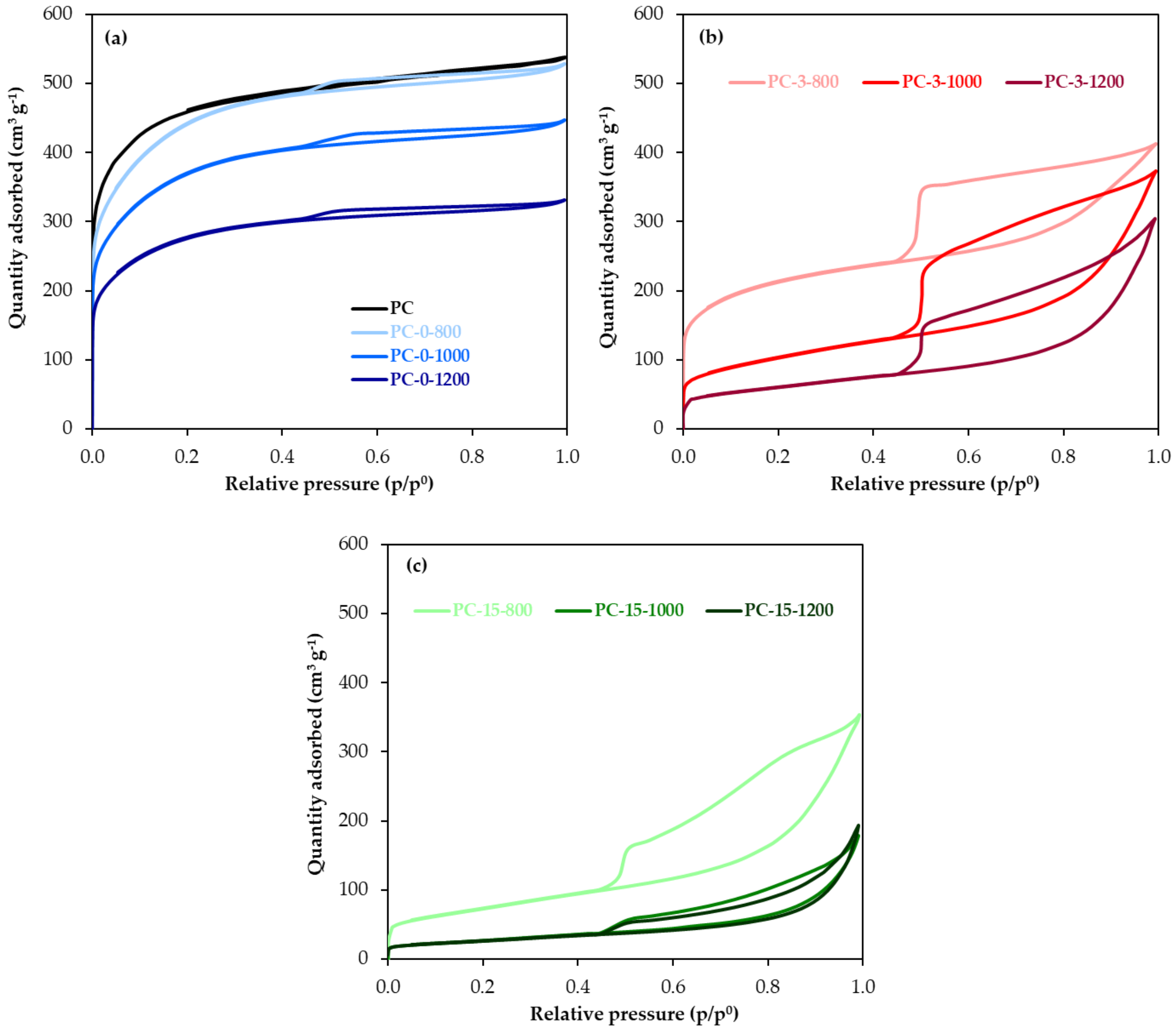

Figure S1. Nitrogen adsorption-desorption isotherms of: (a) PC, PC-0-800, PC-0-1000, PC-0-1200, (b) PC-3-800, PC-3-1000, PC-3-1200, and (c) PC-15-800, PC-15-1000, PC-15-1200 materials.

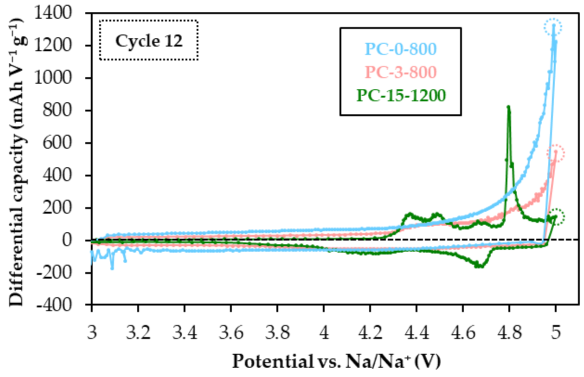

Figure S2. Differential capacity versus potential plot for 12th cycle of PC-0-800, PC-3-800 and PC-15-1200 material-based cathodes at 50 mA g^{-1} in the 2.90-5.00 V vs. Na/Na^{+} potential range.

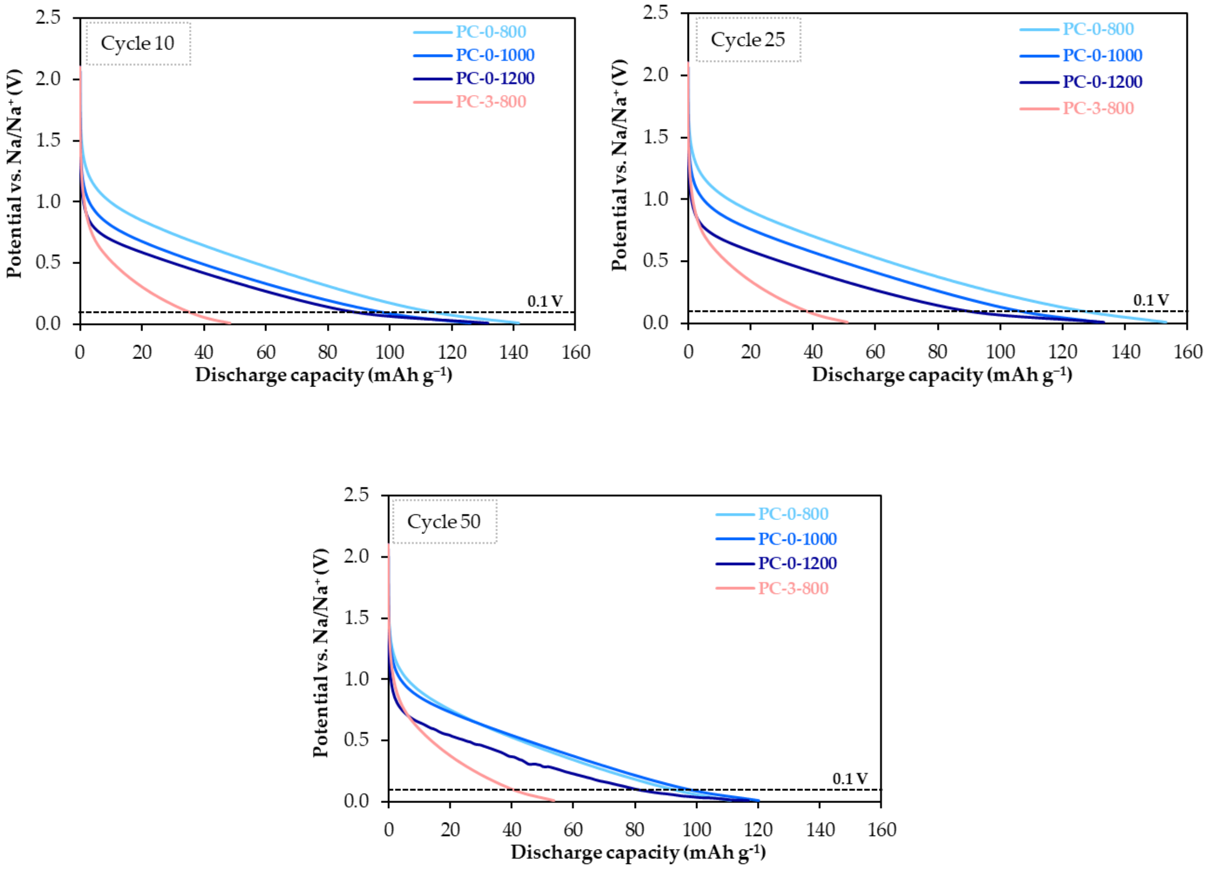

Figure S3. Potential versus discharge capacity plots of PC-0-800, PC-0-1000, PC-0-1200 and PC-3-800 material-based anodes for cycles 10, 25 and 50, in the 2.10-0.01 V vs. Na/Na^{+} potential range.

PC-3-800

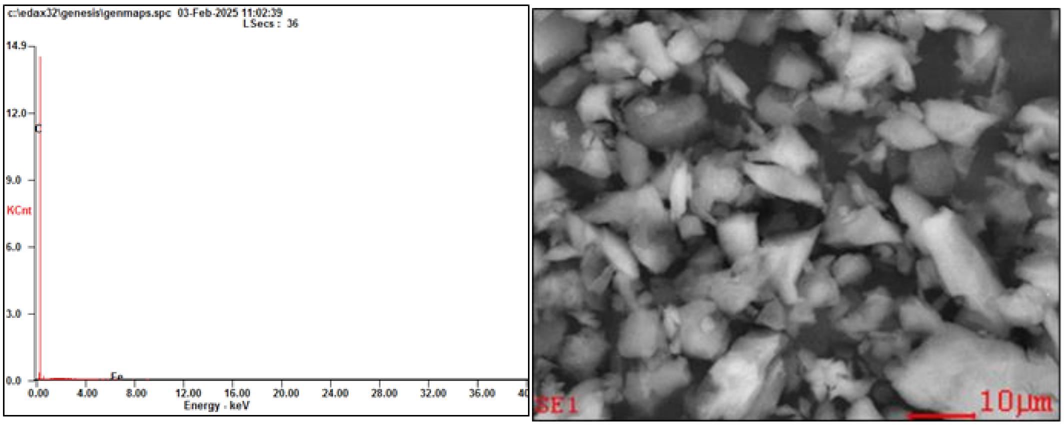

Element	*Wt%*	*At%*
CK	99.82	99.96
FeK	00.18	00.04
Matrix	Correction	ZAF

PC-15-1200

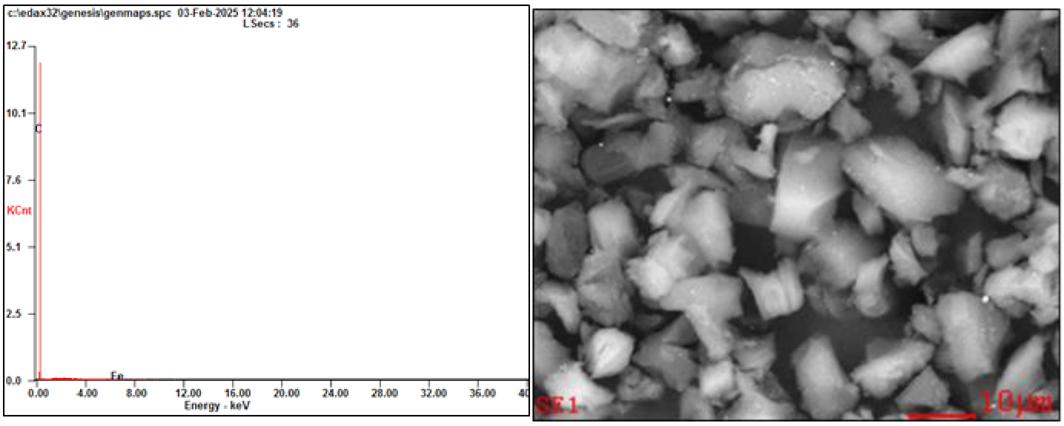

Element	*Wt%*	*At%*
CK	99.51	99.89
FeK	00.49	00.11
Matrix	Correction	ZAF

Figure S4. EDX analysis of PC-3-800 and PC-15-1200 materials.

Table S1. Specific charge capacity (C_{charge}) in the 1st, 2nd, 10th, 20th, 50th, 80th cycles and corresponding coulombic efficiency (CE), and irreversible capacity in the 1st cycle (C_{irr}) from the galvanostatic cycling in 2.9-5.0 V vs. Na/Na^+ potential range at 50 mA g^{-1} for PC-0-800, PC-3-800 and PC-15-1200 material-based cathodes.

**Material**	**C_{charge} 1st cycle (mAh g^{-1})**	**C_{irr}[a] 1st cycle (%)**	**C_{charge} 2nd cycle (mAh g^{-1})**	**CE[b] 2nd cycle (%)**	**C_{charge} 10nd cycle (mAh g^{-1})**	**CE[b] 10nd cycle (%)**	**C_{charge} 20nd cycle (mAh g^{-1})**	**CE[b] 20nd cycle (%)**	**C_{charge} 50nd cycle (mAh g^{-1})**	**CE[b] 50nd cycle (%)**	**C_{charge} 80nd cycle (mAh g^{-1})**	**CE[b] 80nd cycle (%)**
PC-0-800	548	71	417	36	307	41	186	52	59	77	25	88
PC-3-800	265	64	180	52	151	57	147	55	143	52	155	40
PC-15-1200	235	65	124	69	110	81	110	81	105	84	102	84

[a] Irreversible capacity (%) = [C_{charge} (1st cycle) – C_{disc} (1st cycle)] [C_{charge} (1st cycle)]$^{-1}$ x 100

[b] Coulombic efficiency, cycle *i* (%) = [C_{disc} (*i* cycle)] [C_{charge} (*i* cycle)]$^{-1}$ x 100

Table S2. Specific discharge capacity (C_{dis}) in the 1st, 2nd, 10th, 20th, 50th, 80th cycles and corresponding coulombic efficiency (CE),and irreversible capacity in the 1st cycle (C_{irr}) from the galvanostatic cycling in 2.10-0.01 V vs. Na/Na^+ potential range at 50 mA g^{-1} for PC-0-800, PC-0-1000, PC-0-1200 and PC-3-800 material-based anodes.

Material	C_{dis} 1st cycle (mAh g^{-1})	C_{irr}[a] 1st cycle (%)	C_{dis} 2nd cycle (mAh g^{-1})	CE[b] 2nd cycle (%)	C_{dis} 10nd cycle (mAh g^{-1})	CE[b] 10nd cycle (%)	C_{dis} 20nd cycle (mAh g^{-1})	CE[b] 20nd cycle (%)	C_{dis} 50nd cycle (mAh g^{-1})	CE[b] 50nd cycle (%)	C_{dis} 80nd cycle (mAh g^{-1})	CE[b] 80nd cycle (%)
PC-0-800	637	83	177	67	145	92	154	92	102	86	22	93
PC-0-1000	704	90	153	52	126	82	133	86	120	94	128	91
PC-0-1200	513	82	148	66	132	87	134	85	119	88	117	86
PC-3-800	409	92	71	46	49	82	53	85	54	89	52	90

[a] Irreversible capacity (%) = [C_{disc} (1st cycle) – C_{charge} (1st cycle)] [C_{disc} (1st cycle)]$^{-1}$ x 100

[b] Coulombic efficiency, cycle *i* (%) = [C_{charge} (*i* cycle)] [C_{disc} (*i* cycle)]$^{-1}$ x 100
